# Is There a Place for Immune Checkpoint Inhibitors in Vulvar Neoplasms? A State of the Art Review

**DOI:** 10.3390/ijms22010190

**Published:** 2020-12-27

**Authors:** Fulvio Borella, Mario Preti, Luca Bertero, Giammarco Collemi, Isabella Castellano, Paola Cassoni, Stefano Cosma, Andrea Roberto Carosso, Federica Bevilacqua, Niccolò Gallio, Chiara Benedetto, Leonardo Micheletti

**Affiliations:** 1 Division of Gynecology and Obstetrics 1, Department of Surgical Sciences, “City of Health and Science University Hospital”, University of Turin, 10126 Turin, Italy; mario.preti@unito.it (M.P.); stefano.cosma@unito.it (S.C.); andrea88.carosso@gmail.com (A.R.C.); fede.bevi23@gmail.com (F.B.); niccolo.gallio@edu.unito.it (N.G.); chiara.benedetto@unito.it (C.B.); leonardo.micheletti@unito.it (L.M.); 2 Pathology Unit, Department of Medical Sciences, “City of Health and Science University Hospital”, University of Turin, 10126 Turin, Italy; luca.bertero@unito.it (L.B.); giammarco.collemi@gmail.com (G.C.); isabella.castellano@unito.it (I.C.); paola.cassoni@unito.it (P.C.)

**Keywords:** immunology, immunotherapy, immune checkpoint inhibitor, vulvar cancer, vulvar squamous cell carcinoma, vulvar melanoma, vulvar Paget’s disease, neuroendocrine tumor, human papilloma virus (HPV), imiquimod

## Abstract

Vulvar cancer (VC) is a rare neoplasm, usually arising in postmenopausal women, although human papilloma virus (HPV)-associated VC usually develop in younger women. Incidences of VCs are rising in many countries. Surgery is the cornerstone of early-stage VC management, whereas therapies for advanced VC are multimodal and not standardized, combining chemotherapy and radiotherapy to avoid exenterative surgery. Randomized controlled trials (RCTs) are scarce due to the rarity of the disease and prognosis has not improved. Hence, new therapies are needed to improve the outcomes of these patients. In recent years, improved knowledge regarding the crosstalk between neoplastic and tumor cells has allowed researchers to develop a novel therapeutic approach exploiting these molecular interactions. Both the innate and adaptive immune systems play a key role in anti-tumor immunesurveillance. Immune checkpoint inhibitors (ICIs) have demonstrated efficacy in multiple tumor types, improving survival rates and disease outcomes. In some gynecologic cancers (e.g., cervical cancer), many studies are showing promising results and a growing interest is emerging about the potential use of ICIs in VC. The aim of this manuscript is to summarize the latest developments in the field of VC immunoncology, to present the role of state-of-the-art ICIs in VC management and to discuss new potential immunotherapeutic approaches.

## 1. Background

Vulvar cancer (VC) is a rare malignancy, representing 4% of gynecological cancers and 0.3% of all newly diagnosed neoplasms. In the United States, the incidence rate is 2.6 new cases per 100,000 women per year, with a median age at diagnosis of 69 years [1]. During the last decades, the incidence of VC progressively increased in many Western countries [2], especially in women aged 50–60 years [3,4]. This change is mainly secondary to changing sexual habits and increasing exposure to HPV infection. In Austria, a 157% increase in VC incidence has been reported in women under 50 years from 1985–1988 to 1994–1997 [5]. In New Zealand, the incidence rose from 2% to 21% over two 10-year periods (1965–1974 vs. 1990–1994) in the same age group [6]. Furthermore, in Denmark, a significant upward trend has been observed in women under 60 years (+1.60% per year between 1978 and 2007) [7], whereas in Italy the total incidence showed a regular and significant reduction mainly due to decreasing incidences in women aged >60 years from 1990 to 2015; conversely, a 1.20%/year increase has been observed in women younger than 60 years [3].

The most common histological type of VC is squamous cell carcinoma (VSCC), accounting for about 80% of cases, followed by basal cell carcinoma, vulvar melanoma and other less frequent subtypes such as vulvar Paget’s disease (VPD), Bartholin gland adenocarcinoma and neuroendocrine tumors and sarcomas [8]. Concerning VSCC, at least two main oncogenic pathways have been identified. The first one recognizes vulvar high-grade squamous intraepithelial neoplasia (VHSIL) as a precursor and is associated with human papilloma virus (HPV) infection in over 80% of cases, mainly HPV 16 (77.2%), HPV 33 (10.6%) and HPV 18 (2.6%) [9]. This HPV-related pathway is more commonly found in younger women, usually in their third to fifth decade of life [10]. Recognized risk factors are smoking, a high number of sexual partners and immunodepression. Overall, although VHSIL is strongly related to HPV infection, only 30% to as low as 4.9% of VSCC are secondary to HPV [11]. The second pathway arises within chronic dermatoses such as lichen sclerosus and lichen planus, and it is typical of older women. Differentiated vulvar intraepithelial neoplasia (dVIN) has been confirmed as a preinvasive lesion and carries a higher risk of progression over a shorter period of time compared to VHSIL [12]. Even though the majority of VSCCs are not HPV-related, dVIN represents only 2%–10% of all vulvar intraepithelial lesions; thus, dVIN is considered a transient lesion, rapidly progressing to invasive malignancy [10]. Regarding genomic alterations, frequent TP53 mutations have been implicated in the development of HPV-negative VSCCs (HPV-negative/TP53-mutated VSCCs). Histologically, HPV-negative tumors are well-differentiated, keratinizing squamous cell carcinomas and tend to have worse disease-specific survival (DSF). Indeed, HPV status has both prognostic and predictive value. An 83% 5-year overall survival (OS) has been reported for HPV-positive VSCC compared to 48% for HPV-negative/TP53-mutated VSCC [13]. HPV-positive VSCC also shows superior disease outcomes when treated with radiotherapy (RT) compared to HPV-negative VSCC and this radiosensitivity should prompt dose de-escalation [14]. The survival benefit seems to be similar to other radiation-treated HPV-related SCCs, such as head/neck and anal cancers [15]. Recently, a third subgroup of HPV-negative VSCCs has been identified with normal p53 expression and NOTCH1 and HRAS mutations (HPV-negative/TP53 wild type VSCC) [16]. This third subgroup has an intermediate 5-year OS of 64% and further studies are needed to fully characterize its molecular landscape.

VC is staged according to the International Federation of Gynecology and Obstetrics (FIGO) staging system which encompasses the lesion dimension, depth of invasion (DOI) and inguinal lymph node involvement [17]. Almost 60% of VCs are diagnosed at an early stage (FIGO stage I/II), whereas 28% present with regional lymph node spread and 6% with distant metastatic disease [1]. Prognosis is strongly dependent on stage; 5-year OS ranges from 85.5% of FIGO I/II cancers to 20.3% of cases with distant metastases.

Treatment also varies according to FIGO stage. FIGO IA tumors (a single lesion < 2 cm in diameter and DOI < 1 mm) are treated by wide local excision without inguinal lymphadenectomy [18] as the risk of nodal metastasis in stage IA is minimal. For tumors with DOI > 1 mm, a modified radical vulvectomy along with lymph node evaluation, either by sentinel lymph node sampling for tumors ≤4 cm or inguino-femoral lymph node dissection, is the standard of treatment. Adjuvant therapies are dependent upon surgical margins and nodal status. Other prognostic factors such as perineural and lymphovascular invasion must be considered for management after surgery [19]. Treatment for advanced-stage disease is highly individualized and consists of multimodal therapies, including neoadjuvant RT and chemotherapy, to possibly avoid exenterative surgery of the primary tumor [20]. Systemic therapy has been proposed, but the overall rarity of VC makes it challenging to perform randomized controlled trials. Furthermore, differences in terms of inclusion criteria and the absence of reliable prognostic/predictive markers are obstacles to the definition of the best drug regimen for each specific situation. Given the high burden of unresectable/metastatic disease and the frequent comorbidities of elderly and frail women, the need for new therapeutic approaches is clear. A recent study assessing the immune infiltrate in different subtypes of VSCC and its impact on prognosis found an association between T cell-highly infiltrated VSCC and favorable clinical outcomes. In particular, prognosis was strongly impacted by activated CD4+ T cell intraepithelial infiltration; the percentage of infiltrated tumors varies widely according to the different tumor subtypes: from 78% of HPV-positive VSCCs, to 60% of HPV-negative/TP53 wild type and 40% of HPV negative/TP53-mutated tumors [21]. This finding suggests that, in highly infiltrated HPV-positive VSCC, T cells could be exploited to induce an immunoresponse against tumor cells and potentially de-escalate current treatment regimens.

Immune checkpoint inhibitors (ICIs) have reshaped the therapeutic and prognostic paradigms of multiple malignancies, such as melanoma [22] and non small cell lung carcinoma [23]. Regarding gynecological cancers, the overall success of ICIs has been so far limited, with some exceptions. For instance, ICIs are highly effective against deficient mismatch repair (dMMR) and microsatellite instability-high (MSI-h) endometrial cancer, leading to the Food and Drug Administration (FDA) approval of pembrolizumab for this indication in 2017 [24]. Later, pembrolizumab was approved in combination with an antiangiogenic drug (lenvatinib) for pretreated, advanced dMMR endometrial carcinoma irrespective of MSI status. In cervical cancer, pembrolizumab was approved in 2018 by the FDA as a single agent in patients with recurrent or metastatic cervical cancer with disease progression after chemotherapy and tumor expression of programmed death-ligand 1 (PD-L1) (combined positivity score (CPS) ≥ 1), but it is not approved in Europe [25]. Most ongoing studies on cervical cancer are in phase II/III and are aiming to confirm these promising preliminary results achieved in locally advanced or metastatic disease. Even if immunotherapy in ovarian cancers is sustained by a strong biological rationale, ongoing trials have not shown significant benefits and clinical results are largely disappointing [26]. Combining immunotherapy with chemotherapy or RT seems to be the most promising strategy, but the right combination and regimen is still to be defined.

The purpose of this review is to discuss the latest developments in the field of immunoncology for VCs. The current knowledge regarding the VC tumor immune microenvironment will be presented, as well as the most recent clinical results regarding the use of ICIs in VC. Finally, potential novel immunotherapeutic approaches will also be discussed.

## 2. Immunopathology of VC

The tumor microenvironment (TME) is characterized by interactions between cancer and other cell populations within the peritumoral extracellular matrix. In this context, the immune system is one of the most important factors modulating the TME. The complex interactions between tumors and host immunity have been extensively studied in the past few decades, identifying peculiar and variable scenarios according to tumor type, patients’ characteristics, the specific sample type (primary or metastatic) and so on.

Usually, the TME is shaped by a wide range of immune cells belonging to both the adaptive and the innate immune system, including macrophages, dendritic cells, mast cells, B cells, natural killer (NK) and T cells (T helper 1 (TH1) and 2 (TH2), regulatory T cells (T reg), cytotoxic T cells (CTL) and natural killer T cells (NKT)). Some of these cell types can exert an antitumoral effect, whereas others can contribute to tumor progression and metastatization. Furthermore, heterogeneity in density and distribution of immune cells may influence clinical and survival outcomes [27,28,29].

### 2.1. Innate Immune System

The innate or natural immune system comprises the inborn immune mechanisms that defend the host from infection by other organisms in a non-specific manner. It includes anatomical, physical and chemical barriers, circulating molecules and cells with specific phagocytic or lytic functions. A limited number of germline-encoded pattern-recognition receptors (PRRs) that recognize invariant pathogen-associated molecular patterns (PAMPs) characterizes the innate immune system. Detection of PAMPs by PRRs leads to the induction of inflammatory responses and the activation of innate host defenses. Phagocytes, NKT cells, complement and inflammatory cytokines represent the key actors in innate immunity [30,31].

The role of the innate immune system in controlling tumor progression is controversial, as some studies suggest a favorable role in controlling tumor growth, whereas others argue otherwise [32,33]. In this context, a key role is played by macrophages, myeloid-derived suppressor cells (MSCs) and professional antigen-presenting cells (APCs). Macrophages are CD68-, CD86-, and CD163-positive monocyte-derived cells that through the activation of toll-like receptors (TLRs) and non-opsonic receptors can deliver pro-inflammatory cytokines as well as phagocytes and degrade pathogens [34]. Usually, the presence of high macrophage infiltration is related to a poor prognosis, as they contribute to the metastatization process, angiogenesis and suppression of TH1 cells’ immune responses [35]. Macrophages are divided into two main categories: classic/inflammatory M1 (CD86+) and the alternatively activated M2 (CD163+ CD206+) [34].

**Figure 1:**
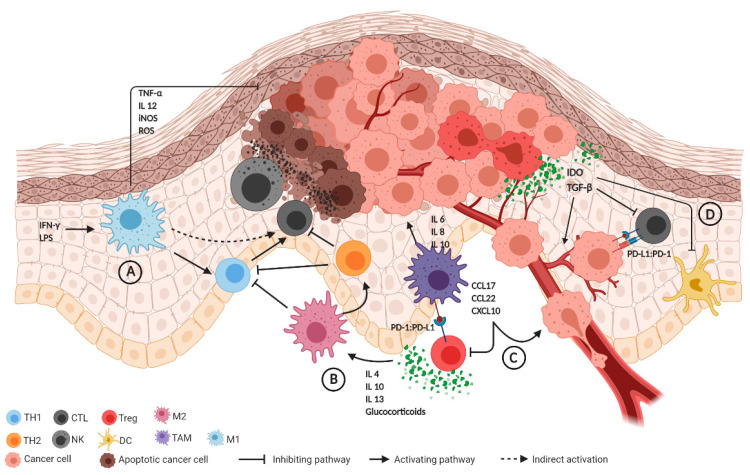
Summary of the immune tumor microenvironment (TME). (**A**) Immune stimuli (e.g., IFN-γ, LPS) activate M1 macrophages that release anti-tumoral agents including TNF-α, IL-12, iNOS and ROS, also promoting the TH1 response. (**B**) Glucocorticoids and IL-4, IL-10, IL-13 stimulate M2 macrophages, which inhibit TH1 and enhance the TH2 response, with subsequent CTL suppression. TH2 activation further inhibits the TH1 response. (**C**) TAMs express PD-L1, CCL17, CCL22 and CXCL10, exerting an immunosuppressive effect through Treg and promoting tumor growth. TAMs are also involved in cancer progression through the release of IL-6, IL-8 and IL-10. (**D**) The release of IDO and TGF-beta by cancer cells inhibits alloreactive lymphocytes. PD-L1 expression by cancer cells also suppresses CTL activity. Abbreviations: CCL17, CCL22: C-C motif chemokine ligand 17 and 22; CTL: cytotoxic T cells; CXCL10: C-X-C motif chemokine ligand 10; DC: dendritic cell; IDO: indoleamine 2,3-dioxygenase; IL 4, 6, 8, 10, 12, 13: interleukin; iNOS: inducible nitric oxygen synthase; LPS: lipopolysaccharide; NK: natural killer; PD-1: programmed death 1; PD-L1: programmed death-ligand 1; ROS: reactive oxygen species; TAM: tumor-associated macrophage; TH1, TH2: T helper 1 and 2; TNF-α: tumor necrosis factor α; TGF-β: transforming growth factor-β; Treg: T regulatory cell.

Classical activation of M1 occurs in response to bacterial molecules (e.g., lipopolysacharide (LPS)) and immune stimuli such as interferon-γ (IFN-γ) (Figure 1A). M1 macrophages are committed to producing tumor necrosis factor α (TNF-α), interleukin-12 (IL-12), inducible nitric oxygen synthase (iNOS), and reactive oxygen species (ROS), all agents with tumoricidal effects (Figure 1A). In addition, M1 macrophages enhance TH1 activity (Figure 1A). On the contrary, M2-activated macrophages exist in different forms depending on the eliciting signals—which include immune suppressive glucocorticoids IL-4, IL-13 and IL-10—and release cytokines that promote a TH2 immune response (Figure 1B) [36]. Tumor-associated macrophages (TAMs) often express the M2 phenotype, but recent evidence suggests that the TAM phenotype is a dynamic condition, supporting a cellular plasticity model. Based on these data, TAMs are able to switch between M1 and M2 phenotypes, sometimes through the coexistence of both M1 and M2 markers, and can vary according to the stage of tumor progression. M1 macrophages are often abundant in chronic inflammatory sites, playing an anti-inflammatory and tumoricidal role; then they switch to an M2-like phenotype, boosting tumor development, angiogenesis and invasiveness. On the other hand, TAMs are involved in cancer progression through the release of specific protumoral cytokines such as interleukin IL-6, IL-8 and IL-10 [37,38,39,40]. TAMs are also able to express immune checkpoint modulators such as PD-L1 and various chemokines (C-C motif chemokine ligand 17 (CCL17), CCL22, C-X-C motif chemokine ligand 10 (CXCL10)) which attract effector/activated T regs that exert an immunosuppressive action and promote tumor growth (Figure 1C) [36,37,38,41,42,43].

In normal vulvar skin stroma, intraepithelial M2 macrophages are the most abundant cell type, whereas myeloid cells are absent. The transition to a pre-neoplastic condition induced by the presence of HPV (dysplasia) is marked by an increase of intraepithelial and stromal M1 and M2 macrophages. Macrophages also play a fundamental role in VSCC development and progression. For example, preclinical studies of HPV-induced oncogenesis using an E6/E7-expressing TC-1 tumor murine model showed that TAM was the predominant population infiltrating neoplastic tissue. Moreover, the depletion of TAMs is linked to an increase in the T cell-mediated anti-neoplastic immune response, leading to a reduction in cancer growth [39]. Additionally in murine models, the addition of an HPV 16-synthetic long peptide (SLP) vaccine to a carbo-platinum plus taxol-based chemotherapy regimen led to a reduction in both circulating and intratumoral myeloid cells and an increase in the T cell response. Similar effects on circulating immune cells were observed in a small cohort of patients who underwent chemotherapy plus vaccination with long peptides mimicking key HPV-16 oncogenic proteins [44]. VSCC is also characterized by a high infiltrate of CD14+ M1 macrophages, which become the dominant population, and the presence of CD14+ cells represents an independent negative prognostic factor for recurrence-free survival (RFS) in HPV-related vulvar intraepithelial neoplasia [45]. A recent large immunohistochemical study performed in 103 patients showed that CD3+ T cells, CD20+ B cells, CD68+ macrophages, forkhead box P3 (FOXP)3+ Treg cells and CD163+ TAMs are present in both peri- and intra-tumoral tissues of VC. Furthermore, the number of CD68+ cells was constantly high in PD-L1-positive tumors and their amount was associated with PD-L1 labeling intensity [46]. Similar results were observed in other studies: Sznurkowski et al. [47] found that an increase in CD56+ and CD68+ cell infiltration was related with the DOI in both p16-positive (a surrogate for HPV infection) and negative VSCC, whereas concerning disease stage and the presence of distant metastases, only the presence of a CD68+ infiltrate in p16 negative tumors showed a positive correlation.

Professional APCs, including dendritic cells (DCs) and Langerhans cells (LCs), represent other key actors in the innate immune response. DCs have a highly effective antigen-presenting ability and are considered to be a critical factor in antitumor immunity. DCs are initiators of adaptive immunity by processing and presenting antigens to T cells and through specific immunomodulatory effects mediated by cell–cell contact and cytokine release. Within the TME, these cells are able to present tumor-associated antigens via the human leukocyte antigen (HLA) class I pathway and this process culminates with the activation of cytotoxic T cells, which results in anti-tumoral activity [48,49]. Regarding LCs, they are a category of APCs commonly found in normal skin and have an immunomodulating action similar to other DCs in TME of SCC [50]. A reduction in the number of LCs was observed in vulvar lichen sclerosus and in VSCC, suggesting that a dysregulation of the skin immune system may lead to suppression of LCs in the vulvar epithelium, promoting carcinogenesis [51].

Concerning the impact of NK/NKT cells on TME in VSCC, Sznurkowski et al. [52] demonstrated that high intraepithelial granzyme B positive infiltrates (which include activated NK, NKT and CTL cells with high cytotoxic activity) were correlated with longer OS in non-metastatic cancer, whereas the number of CTL or CD56+ cells alone were not. Conversely, a correlation between intraepithelial CD56+ (NK/NKT) cells and survival was observed among metastatic VSCC patients, whereas the prognostic significance of granzyme B-dependent killing was not observed. These findings are consistent with the view that NK perforin-mediated lysis is important in the early stage of cancer development, whereas in advanced cancer, NKs, NKTs and CTLs converge to an anergic state. In fact, NKT cells can be classified as type I, also known as invariant NKs, that display different antitumoral weapons, and type II NKTs that, on the contrary, downregulate immunosurveillance. Their relative infiltration in the TME depends on different cytokine concentrations, interaction with mesenchymal stem cells and other factors that are not fully understood [53]. Further investigations about the role of this immune system component could represent a new field of research, paving the way for new treatments [54].

Finally, expression of indoleamine 2,3-dioxygenase (IDO) by VSCC cells is another potential mechanism of immune escape, since it restrains the proliferation of alloreactive T lymphocytes and of DCs through the local reduction of tryptophan, and may partially explain the immunological alterations present in these tumors. Furthermore, the IDO expression in VSCC cells is an independent negative prognostic factor in terms of OS [55,56,57,58].

### 2.2. Adaptive Immune System

Unlike the innate immune response, the adaptive system shows a high specificity for its target antigens [59]. Adaptive responses are based on the antigen-specific receptors expressed on T and B cells: T-cell receptor (TCR) and immunoglobulin (B-cell antigen receptor), respectively.

The main effectors of the adaptive immune response are CD8+ CTL and CD4+ TH lymphocytes. CTL activation requires the interaction with an APC via HLA I and with CD4+ T lymphocytes which have been antigen-primed by the same APC via HLA II. Cancer cells are then eliminated indirectly via complement-mediated antibody cytotoxicity or directly by CTL activity, together with THs [60]. In particular, TH1 cells that are positively selected by M1 macrophages mediate the expansion of tumor specific CTLs from the naïve T pool. By contrast, TH2s, promoted by M2 macrophage, inhibit TH1s, hampering the cell-mediated antitumor response [61]. This unstable equilibrium can be unbalanced in VSCC by different mechanisms of immune evasion and inactivation of the T cell response, such as the high expression of IDO [55], the secretion of transforming growth factor- β (TGF-β) [62] and PD-L1 [46] by cancer cells (Figure 1D) and tumor infiltration by M2 macrophages and T regs [45]. Higher expression of TGF-β in cancer cells is also associated with more advanced cancer stages, possibly leading to metastasis in the regional lymphatic node. This is likely due to the immunosuppressing role of TGF-β in the tumor microenvironment. The TGF-β super-family is a large group of structurally associated proteins including growth and differentiation factors. TGF-β regulates cell growth, apoptosis, differentiation and fibrosis, plays a role in epithelial-mesenchymal transition and can stimulate tumor-associated angiogenesis [63]. TGF-β is secreted by cancer cells and by several other cell types present in the TME, including T regs, macrophages, platelets and fibroblasts. High TGF-β-levels deflect naïve T cell differentiation from the TH1 effector toward the T reg phenotype, and block antigen-presenting functions of DCs [64]. CTLs are activated through direct antigen presentation by major histocompatibility complex (MHC) class I or through T helper cell-mediated activation.

The presence of tumor-infiltrating lymphocytes (TILs) and their prognostic significance in VSCC have been investigated by several authors. A study on 76 paraffin-embedded samples of VSCC found that CD8+ and CD4+ cells were present in both cancer cell nests and stroma, suggesting an immunological synergy, but their presence was not related to better survival [65]. Similar results were observed by De Jong et al., who did not find a relationship between the VSCC prognosis and the number of intratumoral CD8+ T lymphocytes and FOXP3+ T reg lymphocytes. In addition, the authors observed a reduction in the CD8+ lymphocytic infiltrate in tumors expressing HLA class I [66]. These results suggest that T lymphocytes are committed to an anergic state in VSCC. However, in contrast with previous findings, a recent study found a correlation between TILs and prognosis in early-stage surgically treated VSCC. In particular, high intraepithelial infiltration by activated TH lymphocytes (CD3^+^CD8^−^FOXP3^−^) was related to better RFS and OS, independently of HPV and p53 status. Moreover, the percentage of TILs varied between the different VSCC subtypes: HPV-related VSCC was most often strongly infiltrated (78%) followed by the HPV-negative, TP53-wild type VSCC subtype (60%), whereas the lowest T cell infiltration was observed in HPV-negative, TP53-mutated VSCC (40%) [21]. Finally, these authors suggest that the previously reported lack of correlation between TILs and clinical outcomes for VSCC may be due to the high heterogeneity of patients’ clinical characteristics (VSCC etiology, tumor stage, administered treatments) as well as the methodological discrepancies (e.g., differences in terms of analyzed areas or of analyzed T cell populations [21]).

### 2.3. The Role of Immune Checkpoint Regulators

Immune checkpoint regulators are critical modulators of the immune system and play a fundamental role in controlling the tissue damage induced by immunological responses. They are involved in self-tolerance and limitation of autoimmune reactions. Of these molecules, the most studied are the cytotoxic T-lymphocyte-associated protein 4 (CTLA-4) and PD-1 (also known as CD279), which belong to the CD28/CTLA-4 family of co-stimulatory receptors.

PD-1 is a type I membrane protein expressed by immune cells that are chronically activated (predominantly lymphocytes). Following its binding to its ligands (PD-L1 and PD-L2), PD-1 inhibits the proliferation of cytotoxic CD8+ T lymphocytes and modulates the release of pro-inflammatory cytokines. Furthermore, the expression of PD-1 by T regs increases their immunosuppressive ability. Interactions between these PD-1 expressing cells represent the main tumor immune escape mechanism mediated by these pathways in most solid cancers, including gynecological tumors [67,68,69]. A high expression of PD-L1, the main ligand of PD-1, is associated with increased clinical response rates and superior survival outcomes after treatment with ICIs in different tumors such as non small lung cancer and urothelial carcinomas [70,71,72,73]. Several studies investigating oropharyngeal squamous cell carcinomas and cervical cancer have shown that PD-L1 expression is increased in HPV-associated tumors [74,75,76], but these findings are in contrast with what has been observed in VC. In fact, PD-L1 was found to be more frequently expressed in HPV-negative VSCC cells and its expression correlated with tumor aggressiveness, increased inguinal lymph node involvement and poorer prognosis [46]. A recent study performed by Cocks et al. found a worse prognosis for VSCCs with high PD-L1 and CD8 expressions [77], and Lerias et al. confirmed a correlation between PD-L1 levels and nodal involvement [78]. Conversely, a more favorable prognosis was observed by Sznurkowski et al., but only when PD-L1 was expressed by peritumoral immune cells. These authors also observed greater expression of PD-L1 in HPV-negative VSCC cells compared to HPV-positive ones [79]. However, it should be noted that three other studies found high PD-L1 expression in the majority of VSCCs without detecting correlations either with HPV status or prognosis [80,81,82].

Overall, based on the data available so far, the conflicting findings regarding the relationship between HPV status and survival outcomes probably remain controversial due to the heterogeneity of the study cohorts [46,79]. Nevertheless, frequent expression of PD-L1 in VSCC has been consistently reported by many authors [80,81,82], supporting the rationale of ICI-based treatments in VSCC.

### 2.4. Immunological Changes Induced by HPV Infections

**Figure 2:**
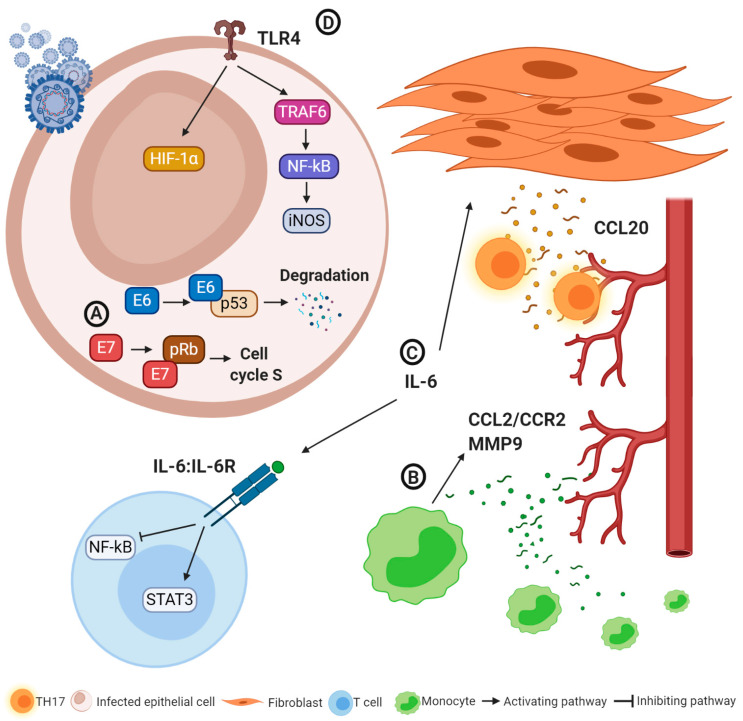
Oncogenic and immune escape mechanisms in human papilloma virus (HPV)-related tumors. (**A**) E6 and E7 proteins contribute to averting apoptosis of infected cells and promoting cell proliferation, paving the way for neoplastic transformation. (**B**) IL-6 reduces the anti-tumoral activity of T lymphocytes and promotes CCL20 expression by cancer-associated fibroblasts and T helper 17 recruitment, also promoting cancer progression. (**C**) CCL2/CCR2 and MMP-9 production lead to monocyte infiltration and angiogenesis. (**D**) TLR4 activation promotes cancer progression through HIF-1α induction and nitric oxygen synthesis. Abbreviations: CCL2 and 20: C-C motif chemokine ligand 2 and 20; CCR2: C-C chemokine receptor type 2; HIF-1α: hypoxia-inducible factor-1α; IL 6: interleukin 6; IL6R: interleukin 6 receptor; iNOS: inducible nitric oxygen synthase; MMP-9 matrix-metalloproteinase 9; NFkB: nuclear factor kappa-light-chain-enhancer of activated B cells; pRB: retinoblastoma protein STAT3: signal transducer and activator of transcription; TH17: T helper 17; TLR4: toll-like receptor 4, TRAF6: tumor necrosis factor receptor-associated factor 6.

HPV infections are related to various pathologies of the lower genital tract, ranging from benign warts to low- and high-grade intraepithelial neoplasms and invasive tumors, with differences related to the specific viral genotypes [83,84]. Based on DNA classification, 15 HPV subtypes (16, 18, 31, 33, 35, 39, 45, 51, 52, 56, 58, 59, 68, 73 and 82) are considered to be at high risk for the development of invasive tumors and their precursor lesions [85]. High-risk HPV infection is responsible for a significant, although minor (less than 40%) proportion of all VCs [11,86]. After the HPV has entered the host cell, it leads to the synthesis of two proteins called E6 and E7 which respectively bind p53 (mediating its degradation and thus preventing apoptosis) and retinoblastoma (Rb) (a protein involved in the regulation cell cycle) proteins, causing uncontrolled cell division (Figure 2A). The accumulation of genetic alterations enabled by this process allows the tumor cell to acquire immune escape mechanisms [25].

The immune response plays a fundamental role in clearing most HPV infections. In fact, if the virus is not effectively eliminated, it can persist for years and promote the transformation from normal epithelium into dysplastic lesions up to invasive tumors. Infections by high-risk HPV are common, but fortunately only a minority of patients develop neoplastic lesions [87]. HPV oncoproteins may also lead to the suppression of acute inflammatory responses, and HPV-positive tumor cells produce only low chemokine levels [84]. Conversely, HPV-transformed cells induce CCL2 production in monocytes, leading to stromal chronic inflammation through two main mechanisms (Figure 2B) [88,89]: (i) the recruitment of additional myelomonocytes via a C-C chemokine receptor type 2 (CCR2)-dependent autocrine mechanism; (ii) the overproduction of matrix-metalloproteinase (MMP)-9, which favors monocyte infiltration and angiogenesis. In the HPV-positive cervical cancer microenvironment, a high production of IL-6 has been observed. IL-6, through the activation of signal transducer and activator of transcription 3 (STAT3) and the suppression of nuclear factor kappa-light-chain-enhancer of activated B cells (NF-kB), promotes the reprogramming of the adaptive immune cell response, resulting in a reduction of the cytotoxic activity of CD8+ T lymphocytes and of the antineoplastic activity of TH1 cells and in an increased expression of MPP-9 [90]. Furthermore, the release of IL-6 activates the C/EBP signaling pathway, leading to CCL20 expression in cancer-associated fibroblasts and TH17 recruitment, which promote tumor progression and angiogenesis (Figure 2C) [91]. It is also known how HPV infection can alter the expression of TLRs and of their signaling pathways, favoring the persistence of the virus. Different types of TLRs can exert an antitumor effect or promote neoplastic progression. For instance, in HPV-related cervical cancer TLRs 1, 2, 3, 4, 5, 6 and 9 are expressed more [92,93]. In particular, the expression of TLR4 in the uterine cervix infected by HPV is associated with cancer development and progression via the overproduction of hypoxia-inducible factor-1α (HIF-1α) [94]. Furthermore, activation of TLR4 leads to a high expression of iNOS through the activation of various genes (tumor necrosis factor receptor-associated factor 6 (TRAF6), mitogen-activated protein kinase (MAPK), NF-Kb), resulting in high levels of nitric oxide, increased HPV infectiveness and tumor development (Figure 2D) [93,95,96,97].

In this context, the introduction of HPV vaccination as a primary prevention strategy has been shown to reduce the incidence of invasive and premalignant cervical lesions among vaccinated women [98]. A recent analysis by the Norwegian Cancer Register estimated promising reductions of HPV-related VSCCs in the coming years thanks to this public health program [99].

Cancer cells infected with oncogenic HPV genotypes develop a series of immune evasion mechanisms; thus, several strategies have been investigated to tackle them by enhancing CD4+ and CD8+ T cell responses such as gene-based, protein-based, peptide-based and dendritic-cell-based vaccines [100]. ADXS11-011 (axalimogene filolisbac) is an irreversibly attenuated *Listeria monocytogenes* (Lm) combined with a nonhemolytic fragment of listeriolysin O (Lm-LLO) and secreting the Lm-LLO-HPV E7 fusion protein directed against HPV-positive cancers [101]. A randomized phase 2 study evaluated its safety and efficacy administered with or without cisplatin in women with recurrent/refractory HPV-positive cervical cancer following prior chemotherapy and/or RT. Combined 12-month and 18-month OS rates of 34.9% (38/109 patients) and 24.8% (27/109 patients) were respectively achieved, resulting in an approximate 1.5- to 2-fold increase in median OS rates compared with the historical GOG series [102].

Recently, another study using the axalimogene filolisbac-based vaccine (ADXS-HPV) in 50 patients with advanced, platinum-refractory cervical cancer, achieved a 12-month OS of 38% (19/50 patients) [103].

## 3. Clinical Role of ICIs in VSCC

The KEYNOTE-028 study is a non-randomized, multicenter, multicohort phase Ib trial on the use of pembrolizumab (a PD-1 inhibitor) in patients (*N* = 474) with 20 different PD-L1-positive advanced solid cancers, including 18 VSCCs. The median OS in patients affected by VSCC was 3.8 months, the objective response rate (ORR) <10% and the median progression-free survival (mPFS) <5%. Within the VSCC cohort, only one patient achieved a partial response (PR), seven had stable disease (SD), whereas six experienced disease progression (PD). The progression-free survival rates were 20% and 7% at 6 and 12 months, whereas the OS rates were 42% and 28%, respectively. Concerning adverse effects (AEs), no stratification by cancer type was made, but most of the trial participants experienced at least one AE, in particular fatigue (35%), nausea (26%), decreased appetite (22%), diarrhea (22%) or constipation (20%). In most of cases a G2 (36%) or G3 (39%) toxicity was reported. In this trial, the efficacy of potential predictive biomarkers was also investigated: the presence of a T cell-inflamed gene-expression profile, PD-L1 expression and higher tumor mutational burden (TMB) resulted the best predictors of responses among the different types of solid tumors. These three biomarkers were observed in 72%, 44% and 17% of VSCC patients respectively [104].

A phase I-II study (the CheckMate 358 trial) evaluated the role of nivolumab (PD-1 inhibitor) in metastatic/recurrent cervical (*N* = 19) and vulvar/vaginal (*N* = 5) cancer. Among vulvar/vaginal tumors a partial responder (HPV-negative VC) was reported and 12-month and 18-month OS rates of 40% and 20% were observed, with a PFS rate of 40% at 6 months [105].

Recently, a recurrent VC characterized by PD-L1 and PD-1 mutations (PD-1 positive cells >5/high-power field (HPF) and 2+ PD-L1 positivity, high mutational load) was successfully treated with pembrolizumab: after two cycles of immunotherapy a clinical remission was observed, which was then confirmed by a CT scan after six cycles, which showed a partial response according to Response Evaluation Criteria in Solid Tumours (RECIST) criteria [106].

More recently, a single-arm phase II clinical study of pembrolizumab combined with cisplatin and RT for women with unresectable locally advanced or metastatic VC was proposed. This study is currently underway and recruiting patients [107]. Several pieces of data support the use of RT in combination with ICIs. RT can trigger the expression of PD-L1 and PD-1 on tumor cells, making them targets for ICIs [108]. Furthermore, preclinical studies on murine models showed that RT plus ICIs leads to an increase in CD8+ TILs, a decrease in T reg lymphocytes and in MSCs, and in the upregulation of MHC class I, favoring the activity of ICIs [107].

A summary of the reported data regarding the use of ICIs in VC is presented in Table 1.

## 4. Potential Role of ICIs in Rare Vulvar Tumor Types

### 4.1. Vulvar Melanoma

Melanoma is a rare neoplasm (1% of all skin cancers) with a significant clinical impact due to its aggressive behavior and poor prognosis. Vulvar melanoma represents a rare variant of the cutaneous melanoma. Classically, this tumor has been classified as a mucosal melanoma, but recent molecular profiling data suggest that melanomas of the lower genital tract represent a distinct category compared to other mucosal or cutaneous melanomas [111,112]. Current evidence concerning its management strategies and therapeutic approaches relies on small retrospective series and extrapolation from skin melanoma guidelines. In fact, there is no real consensus about the treatment of this neoplasm and prognosis is poorer compared to melanomas of other sites [113,114,115].

Melanomas are among the most immunogenic tumors and the development of ICIs represented a turning point in the treatment of advanced melanoma. Combined targeting of CTLA-4 (ipilimumab) and PD-1 (nivolumab, pembrolizumab) allowed researchers to achieve an OS of 50% in eligible patients. Furthermore, a substantial fraction of long-term survivors seems to remain disease-free even after discountinuation of treatment. In fact, ICI-based immunotherapy is approved by the FDA as a standard treatment for patients with advanced or recurrent melanoma [116,117,118].

The TME of vulvar melanoma is poorly understood. In cutaneous melanoma, the presence of CD8+ TILs is related to a better prognosis and the CD8+ T-cell density is a predictive marker of response to PD-1 blockade [119,120], whereas infiltration by FOXP3+ T regs has been shown to correlate with a worse prognosis [121]. Some studies [122,123] suggest an association between PD-L1 expression and a worse prognosis in melanoma, but its role remains controversial [124]. A recent study [123] investigated the role of immune TME in vulvar melanoma—75 women with vulvar melanomas were included; of these, 21 of 75 (28%) and 39 of 75 (52%) developed local recurrence and distant metastasis, respectively, and the death rate was 33% (median follow-up 26 months). A membranous and/or cytoplasmic PD-L1 expression by ≥ 5% of tumor cells was observed in 17 patients (23%). This marker (*p*-value = 0.04), as well as a higher number of peritumoral FOXP3+ lymphocytes (*p*-value = 0.004), was found to be significantly associated with better survival. This finding is controversial and conflicts with our knowledge regarding cutaneous melanoma [121,125]. Vulvar melanoma probably represents a category of its own, compared to other subtypes of melanocytic tumors. Another study on 14 vaginal and 37 vulvar melanomas showed high expression of both PD-L1 (56%) and PD-1 (75%) [111]; a high expression of PD-L1 was also reported in other series [126,127], paving the way for implementing ICIs in these tumors.

Focusing on mucosal melanomas in a small cohort of five patients affected by head and neck mucosal melanoma, PD-L1 expression by tumor cells was <5% in all cases and no clinical response was achieved by systemic therapy with PD-1 inhibitors [128]. A pooled analysis took into account 889 patients (including 86 mucosal melanomas and 665 cutaneous melanomas) who received nivolumab alone and 361 patients who received nivolumab plus ipilimumab (including 35 mucosal melanomas and 326 cutaneous melanomas). In patients treated with nivolumab alone, median progression-free survivals (PFS) of 3.0 months (95% CI, 2.2–5.4 months) and 6.2 months (95% CI, 5.1–7.5 months) with an ORR of 23.3% (95% CI, 14.8–33.6%) and 40.9% (95% CI, 37.1–44.7%) for mucosal and cutaneous melanoma were obtained, respectively. The median PFS in patients treated with the nivolumab plus ipilimubab combinatiob was 5.9 months (95% CI, 2.8 months to not reached) and 11.7 months (95% CI, 8.9 to 16.7 months) for mucosal and cutaneous melanoma, with ORRs of 37.1% (95% CI, 21.5–55.1%) and 60.4% (95% CI, 54.9–65.8%), respectively. These results suggest the greater efficacy of combined treatment and overall worse survival outcomes for mucosal melanomas [129]. A recent systematic review focused on the clinical outcomes of advanced mucosal melanomas treated with ICIs—patients who received nivolumab or pembrolizumab alone achieved an ORR higher than 15% and a median OS higher than 11 months. Patients treated with an anti-CTLA-4 plus anti-PD-1 combination achieved an ORR > 33%, and interestingly anti-PD-1 plus RT showed an ORR > 50% [130].

Regarding low genital tract melanomas, two recurrent cases (cervical and vaginal melanomas) treated with nivolumab have been recently reported and both patients achieved disease remission [131], whereas treatment of a cervical melanoma with pembrolizumab did not prove effective [132]. In a small cohort of six patients with metastatic vulvar/vaginal melanomas treated with an anti-CTLA-4 ICI, disease progression was observed in four patients, a stable disease in one patient (maintained for 11 months) and an important clinical response was obtained in one patient, achieving long survival (31 months). The 1-year survival rate was 33% [109] (Table 1). As previously mentioned, the RT plus ICIs combination can lead to important results when dealing with advanced neoplasms. In a series of three vaginal and a cervical melanomas treated with ipilipumab plus RT, a complete response (CR) was observed in all women after completion of treatment. Later, two patients developed distant metastasis at 9 and 10 months after diagnosis, whereas two remained disease-free at 20 and 38 months [133].

### 4.2. Vulvar Paget’s Disease

VPD is an extremely rare skin neoplasm, mainly affecting postmenopausal women, defined by the World Health Organization (WHO) as “an intraepithelial neoplasm of epithelial origin expressing apocrine or eccrine glandular-like features and characterized by distinctive large cells with prominent cytoplasm, referred to as Paget cells” [134]. In 2001, Wilkinson and Brown [135] proposed a classification distinguishing two different subtypes—primary cutaneous VPD and secondary non-cutaneous VDP developing from a digestive or urinary tumor. Cutaneous VPD can also be classified according to the DOI—in situ (the most common form), micro-invasive (DOI ≤ 1 mm) and invasive (DOI > 1 mm) [136].

VPD is characterized by an indolent clinical course, although the diagnosis is often late and treatment unsatisfactory due to high relapse rates. In addition, the invasive forms are much more aggressive than in situ and micro-invasive tumors and are characterized by a worse survival [137,138,139,140]. The main therapy consists of an aggressive surgical excision of which the extension depends on the specific disease spread and on patients’ characteristics, but regardless of its type this therapy is burdened by a high rate of recurrence [137,138,140,141].

Some data are available regarding the immune TME of VPD. A study evaluated the presence of FOXP3+ cells in 29 primary and 13 recurrent VPD tissue samples. T regs were frequently observed at the epidermal-dermal junction, surrounded by skin without inflammatory infiltrates and, interestingly, the authors observed a correlation between the number of T regs, the positivity of the surgical margins and a higher recurrence rate [142]. The number of CD4+ CD22+ FOXP3+ T regs was higher in invasive extramammary PD (EMPD) compared to in situ EMDP, whereas high numbers of CD163^+^ macrophages and high MPP-9 expression were detected only in invasive EMDP [143]. Furthermore, the activation of the receptor activator of nuclear factor kappa-Β (RANK)/RANK ligand pathway by the interaction between RANK+ macrophages and Paget cells expressing RANK ligand contributes to the development of an immunosuppressive environment, emphasizing once again the ability of tumors to escape the cytotoxic immune response [144]. More recently [145], an increase in intraepithelial CD8+ and FOXP3 cells was observed in patients with VPD treated with local application of imiquimod, an immunomodulatory drug targeting TLR7 and leading to TH cell activation [146].

The expression of PD-L1 in Paget disease has only been investigated in recent years. A recent analysis [147] of 21 EMPD cases, including 5 VPD cases, found that PD-L1 was expressed only by three tumors, whereas PD-L1 expression within the tumor-associated immune infiltrates was observed in 15 EMPD cases. Another study [148] of 48 patients with EMPD (25 VPD) confirmed that tumor cells rarely express PD-L1, whereas PD-L2 expression was not observed in any tumor. PD-L2 was expressed occasionally by leukocytes and PD-L1 was focally detected in TILs. The majority of cutaneous EMPDs showed high levels of B7 family members (B7-H3 and B7-H4), melanoma-associated antigen (MAGE-A) and New York esophageal squamous cell carcinoma 1 (NY-ESO-1) in cancer cells in both in situ and invasive forms—currently, dozens of clinical trials evaluating drugs directed against these molecules are underway for various tumor types, but unfortunately none of these studies is focused on EMPD [148]. Low expression of PD-1 by cancer cells was also noted by another study including 13 VPD and five scrotal Paget’s disease cases, but intriguingly EMPDs showed a high tumor mutational burden (≥10 mutations/Mb) [149]. In 22 EMPD cases, no expression of PD-L1 was detected in cancer cells, but occasionally CTLA-4 expression was observed among the tumors [150]. Finally, a large study based on samples of 41 women affected by VPD found a positive PD-L1 expression in 10% of in situ VPDs and in 27% of invasive-VPDs [151]. Curiously, MSI was not observed by most authors [149,151,152], however a single study found that 8/20 EMPDs harbored germline mutations of mismatch repair genes (MMR), five of which exhibited MSI [153].

There are currently no clinical data on the use of ICIs for treating VPD; however, a recent, ongoing study on the role of nivolumab and ipilimumab in the treatment of rare malignancies is including EMPD and VC cases (NCT02834013).

### 4.3. Neuroendocrine Tumors

Neuroendocrine tumors (NETs) are a heterogeneous group of neoplasms that arise from aggregates of endocrine cells present in different organs, most frequently the lungs or gastrointestinal tract. NETs are relatively rare tumors, representing less than 0.5% of all malignant tumors, although their diagnosis has increased in recent years, probably due to improved awareness. Most of these tumors are benign, but 12% to 22% present with distant metastases [154,155].

In the female genital tract, NETs are extremely rare. Data from the Surveillance, Epidemiology and End Results (SEER) registry from 1987 to 2012 showed just 559 cases of gynecological NETs and only 39 of them involved the vulva/vagina [156]. Conversely, the most common site is the ovary for benign carcinoids (often arising within a dermoid tumor) and the cervix for high-grade NETs [157]. Indeed, vulvar/vaginal NETs are extremely rare and usually consist of high-grade neuroendocrine carcinomas, whereas the vulva can also be affected by Merkel cell carcinoma [157]. Most of the data concerning the efficacy of ICIs in NETs derive from gastrointestinal tract neoplasms and small cell lung cancers. Data on 107 patients affected by heavily pretreated NETs from the KEYNOTE-028 study (NCT02054806) suggested that pembrolizumab alone has limited efficacy in this setting (median OS (95% CI) had not been reached (18.8–not reached), whereas the 6-month OS rate was 84.6%). Sixty-one patients achieved SD as the best response and curiously four CRs were observed among patients with PD-L1-negative tumors [110]. Recently, an ORR of 25% was achieved in a phase II basket trial including 32 NETs (18 high-grade and 14 low/intermediate grade) treated with the nivolumab plus ipilimumab combination—the response rate was 8/18 (44%) among high-grade NETs, whereas no response was observed in low/intermediate NETs [158]. An open-label, phase II basket trial of pembrolizumab in women with recurrent small cell NETs of the lower genital tract (six cervical NETs and one vulvar NET) showed minimal activity in this subset of patients: after 27 weeks, only one patient had stable disease, whereas six experienced progression [159], suggesting a limited role for this drug in this setting, as already reported by Strosberg et al. [110] (Table 1). Interestingly, a patient with metastatic, chemo-resistant cervical large cell NET showed a near CR to nivolumab plus stereotactic body RT, suggesting again a role for the ICIs plus RT combination [160].

Regarding Merkel cell carcinoma, important results have been obtained with the use of ICIs; indeed, the FDA has approved avelumab and pembrolizumab for their treatment, whereas the European Medicine Agency has approved avelumab only [155], but unfortunately results concerning vulvar Merkel cell carcinomas have never been reported [161,162,163,164].

## 5. Conclusions and Future Directions

VCs are characterized by a series of immune escape mechanisms that confer aggressiveness and lead to poor clinical outcomes. Currently, data on the role of ICIs in VCs are derived from case reports or limited series, often extrapolated from trials involving other types of solid tumors without a careful selection of patients. Based on these data, treatment with single ICIs has shown modest results overall, although interesting cases of disease remission were observed when ICIs were combined with RT. In fact, the evidence, although limited, supports the efficacy of this combination in gynecological cancers [165,166] and two trials on VSCC and vulvar melanoma are ongoing [107,133].

Another intriguing mechanism is represented by the synergistic effect between HPV vaccines and ICIs. Recently, a single-arm phase 2 clinical trial on recurrent HPV 16-related tumors (22 oropharyngeal, one cervical and one anal) using ISA 101, a HPV 16-SLP vaccine in combination with an anti-PD-1 agent (nivolumab), obtained an encouraging median survival of 17.5 months, suggesting a benefit of this kind of combined approach [167]. Another combination that could play a role in the treatment of VCs (in particular VPD) is represented by imiquimod plus ICIs. In selected patients, imiquimod combined with other treatments has been shown to be effective in the local control of skin metastases from melanoma [168] and breast cancer [169,170]. Finally, some reports show a benefit of imiquimod-plus-ICIs combinations in advanced melanomas [171,172].

Therefore, is there a place for ICIs in VC treatment? Probably yes. ICI monotherapy has not yielded striking results, but their combination with other agents represents a promising avenue. Patients with VC at high risk of recurrence or with metastatic/relapsed disease should be treated at referral institutions with high caseloads and specific experience in the management of these tumors and ideally should be included in well-designed, multicentric clinical trials. Specific attention should also be paid to investigating novel potential prognostic and predictive markers that may affect the response to ICIs and other immunotherapies (HPV status, characteristics of immune cells infiltrate, expression of specific markers and the presence of genomic or molecular signatures).

## Figures and Tables

**Table 1:** Studies and case reports exploring the role of Immune checkpoint inhibitors (ICIs) in recurrent/advanced vulvar cancers (VCs).

Type of Study	Antibody	Clinical Setting	Number of Patients	Outcomes	Reference
KEYNOTE-028 Non-randomized, multicenter, multicohort phase Ib trial	Pembrolizumab (PD-1 inhibitor)	Avanced tumors	18 VSCC out of 474 tumors	PFS was 20% and 7% at 6 and 12 months; OS rate was 42% and 28% at 6 and 12 months	Ott et al. [104]
CheckMate 358 trial Phase I-II study	Nivolumab (PD-1 inhibitor)	Metastatic/recurrent tumors	19 cervical tumors, 5 vulvar/vaginal tumors	In vulvar/vaginal cohort: OS was 40% and 20% at 12-month and 18 month OS; PFS was 40% at 6 months	Naumann et al. [105]
Case report	Pembrolizumab (PD-1 inhibitor)	Recurrent VC with PD-L1 and PD-1 mutation	1	Near CR at 6 months of treatrment	Shields et al. [106]
Single-arm phase II clinical study	Pembrolizumab (PD-1 inhibitor)	Unoperable locally advanced or metastatic tumors	Recruiting. Target enrollement: 24 patients.	Primary endpoint: 95% CI for ORR. Secondary endopint: RFS-6	Yeku et al. [107]
Retrospective series	Anti-CTLA-4	Metastatic vulvar-vaginal melanomas	6	33% survival rate at 1 year	Quéreux et al. [109]
Open-label, phase II basket trial	Pembrolizumab (PD-1 inhibitor)	Recurrent small cell NET	6 cervical, 1 vulvar NETs	Median OS not achieved (18.8-not reached) OS 84.6% at 6 months	Strosberg et al. [110]

**Abbreviations:** CR: complete response; CTLA-4: cytotoxic T-lymphocyte-associated protein 4; ICIs: immune checkpoint inhibitors; NET: neuroendocrine tumors; OS: overall survival; PD-1 programmed death 1; PD-L1: programmed death ligand 1; PFS: progression-free survival; VC: vulvar cancer; VSCC: vulvar squamous cell carcinoma.

## Abbreviations

AEs	adverse effects
APC	antigen-presenting cell
CCL	C-C motif chemokine ligand
CCR-2	C-C chemokine receptor type 2
CPS	combined positivity score
CR	complete response
CTL	cytotoxic T cells
CTLA-4	cytotoxic T-lymphocyte-associated protein 4
CXCL	C-X-C motif chemokine ligand
DCs	dendritic cells
dMMR	deficient mismatch repair
DOI	depth of invasion
DSS	disease-specific survival
dVIN	differentiated vulvar intraepithelial neoplasia
EMPD	extramammary Paget’s disease
FDA	Food and Drug Administration
FIGO	International Federation of Gynecology and Obstetrics
FOXP3	forkhead box P3
HIF-1α	hypoxia-inducible factor-1α
HLA	human leukocyte antigen
HPF	high-power field
HPV	human papilloma virus
ICI	immune checkpoint inhibitor
IDO	indoleamine 2,3-dioxygenase
IL	interleukin
ILR	interleukin receptor
INF-γ	interferon-γ
iNOS	inducible nitric oxygen synthase
LCs	Langerhans cells
*Lm*-LLO	*Listeria monocytogenes*-listeriolysin lipopolysaccharide
LPS	lipopolysaccharide
MAGE-A	melanoma-associated antigen
MAPK	mitogen-activated protein kinase
MHC	major histocompatibility complex
MMR	mismatch repair
MMP-9	matrix-metalloproteinase 9
mPFS	median progression-free survival
MSCs	myeloid-derived suppressor cells
NET	neuroendocrine tumor
MSI-h	microsatellite instability-high
NF-kB	nuclear aactor kappa-light-chain-enhancer of activated B cells
NK	natural killer
NKT	natural killer T cells
NY-ESO-1	New York esophageal squamous cell carcinoma 1
ORR	objective response rate
OS	overall survival
PAMPs	pathogen-associated molecular patterns
PD	progression disease
PD-1	programmed death-1
PD-L1	programmed death-ligand 1
PD-L2	programmed death-ligand 2
PFS	progression-free survival
PR	partial response
PRRs	pattern-recognition receptors
RANK	receptor activator of nuclear factor kappa-Β
Rb	retinoblastoma
RECIST	Response Evaluation Criteria in Solid Tumours
ROS	reactive oxygen species
RFS	recurrence-free survival
RT	radiotherapy
SD	stable disease
SEER	Surveillance, Epidemiology and End Results
SLP	synthetic long peptide
STAT3	signal transducer and activator of transcription 3
TAMs	tumor-associated macrophages
TGF-β	transforming growth factor-β
TH	T helper
TH1	T helper 1
TH2	T helper 2
TILs	tumor infiltrating lymphocytes
TLR	toll-like receptors
TMB	tumor mutational burden
TNF-α	tumor necrosis factor α
TRAF6	tumor necrosis factor receptor-associated factor 6
T reg	regulatory T cells
VC	vulvar cancer
VHSIL	vulvar high-grade squamous intraepithelial neoplasia
VPD	vulvar Paget’s disease
VSCC	vulvar squamous cell carcinoma
